# Phylogenomic and super‐pangenome analyses unveil the genetic landscape of tomato evolution and domestication

**DOI:** 10.1111/pbi.70199

**Published:** 2025-06-15

**Authors:** Jingyin Yu, Qionglin Chen, Lu Yuan, Shouli Feng, Miaomiao Huang, Peng Zheng, Guang Chen, Xiaoyuan Tao, David Edwards, Zhong‐Hua Chen, Shengchun Xu

**Affiliations:** ^1^ Xianghu Laboratory Hangzhou China; ^2^ Institute of Digital Agriculture Zhejiang Academy of Agricultural Science Hangzhou China; ^3^ Centre for Applied Bioinformatics and School of Biological Sciences University of Western Australia Perth WA Australia; ^4^ School of Agriculture, Food and Wine, Waite Research Institute University of Adelaide Penrith SA Australia

**Keywords:** gene‐based super‐pangenome, genetic diversity, agronomic traits, presence/absence variation (PAV), crop improvement, sustainable production

## Abstract

The tomato (*Solanum lycopersicum* L.), a principal fruit crop, exhibits significant genetic diversity shaped by domestication and breeding. Analysis of the gene‐based super‐pangenome, a catalogue of all genes across diverse genome‐sequenced tomatoes, has not yet been fully explored. Here, we present a comprehensive analysis of the gene‐based super‐pangenome across 61 genetically diverse tomato varieties, revealing 59 066 orthologous groups, thereby providing a detailed genetic framework for understanding the evolution of tomatoes. Our phylogenetic analysis recalibrates the position of *S. galapagense*, challenging existing paradigms of tomato evolution. Identification of genes linked to key agronomic traits such as fruit size, ripening and stress tolerance, along with their presence/absence variation among accessions, offers a rich source of genetic markers for breeding programs. The study also highlights the impact of whole‐genome triplication (WGT) and tandem gene duplication (TD) events on gene family expansion, particularly in distant wild relatives. The analysis of the LRR‐RLK gene family, important for plant development and defence, reveals substantial sequence diversity and conservation. Rapidly evolving genes and those under positive selection, such as HAI3, CYP711A1/MAX1, WRKY9 and CNGC15, are implicated in stress tolerance and defence mechanisms. The identification of these genes, along with specific pathogenesis‐related genes in distant wild relatives, suggests potential strategies to improve fruit shelf life, fruit set and stress tolerance in elite tomato cultivar breeding. Additionally, we have developed the tomatoPangenome platform, integrating genomic and pangenomic data, gene families and tools, to support sustainable production of high‐quality, climate‐resilient tomatoes and advance selective breeding for future food security.

## Introduction

Tomato (*Solanum lycopersicum* L.), one of the most widely cultivated fruit crops worldwide, belongs to the genus *Solanum* in the Solanaceae family (Quinet *et al*., 2019). There are many wild relatives in the tomato clade with either red or green mature fruits, which exhibit large variation in flavour and nutritional properties (Ercolano *et al*., 2021). The blueberry‐sized *S. pimpinellifolium* (SP) is the ancestor of cultivated tomatoes (Rick and Fobes, 1975; Zuriaga *et al*., 2009), while the early domesticated cherry‐sized *S. lycopersicum* var. *cerasiforme* (SLC) is the evolutionary intermediate between SP and the large‐fruited modern tomato, *S. lycopersicum* var. *lycopersicum* (SLL) (Blanca *et al*., 2012; Jenkins, 1948; Rick and Fobes, 1975). Thus, it was proposed that cultivated tomatoes have been subjected to two phases of domestication: the domestication from SP to SLC and the improvement from SLC to SLL (Lin *et al*., 2014), leading to major changes in fruit shape, taste, yield and quality (Blanca *et al*., 2012, 2015). Among the large‐fruited SLL, there are early cultivars (vintage), modern domesticates and modern cultivars (fresh market), indicative of breeding goals that have targeted large‐fruited tomatoes (Alonge *et al*., 2020; Blanca *et al*., 2015). Notably, the evolution and domestication of tomato species has been accompanied by changes in gene dosage and function that gave rise to variation in important traits or phenotypes among various tomato accessions (Gao *et al*., 2019).

Comparative analysis of the genomes of multiple tomato accessions, including distant wild relatives, ancestral wild species, early domesticates and modern cultivars, has revealed many functional genes and gene families associated with physiological and morphological traits (Li *et al*., 2023; Sato *et al*., 2012; Schmidt *et al*., 2017). Although a reference crop genome is a good starting point to assess the basis of complex agronomic traits, a gene‐based super‐pangenome provides insights into the genetic diversity of a crop and its relatives. Several tomato pan‐genomes, consisting of various tomato accessions, have been reported, and multiple genes encoding commercially important traits have been identified at the pan‐genome level (Gao *et al*., 2019; Li *et al*., 2023; Zhou *et al*., 2022). Studies using a pan‐genome constructed with a reference genome from SLL (Heinz 1706, SL3.0) and a non‐reference genome assembled from 725 representative tomato accessions (Gao *et al*., 2019) revealed that many of the lost or negatively selected genes are related to disease resistance. Another pan‐genome was generated with 32 reference‐level genome assemblies to help identify genes and alleles related to agronomic traits by resolving the heterogeneity of genomic sites (Zhou *et al*., 2022), while a recent study reported a tomato super‐pangenome (Li *et al*., 2023). However, reports and comprehensive databases on pan‐genome gene repertoires and analysis of the functional pan‐genes related to agronomic traits remain limited in tomatoes. Here, we present the gene repertoire of a tomato gene‐based super‐pangenome and the tomatoPangenome platform (http://www.varnatech.cn/tomatoPan/) containing 61 wild ancestral, domesticated and cultivated tomatoes.

## Results

### High‐quality tomato gene‐based super‐pangenome

**Figure 1:**
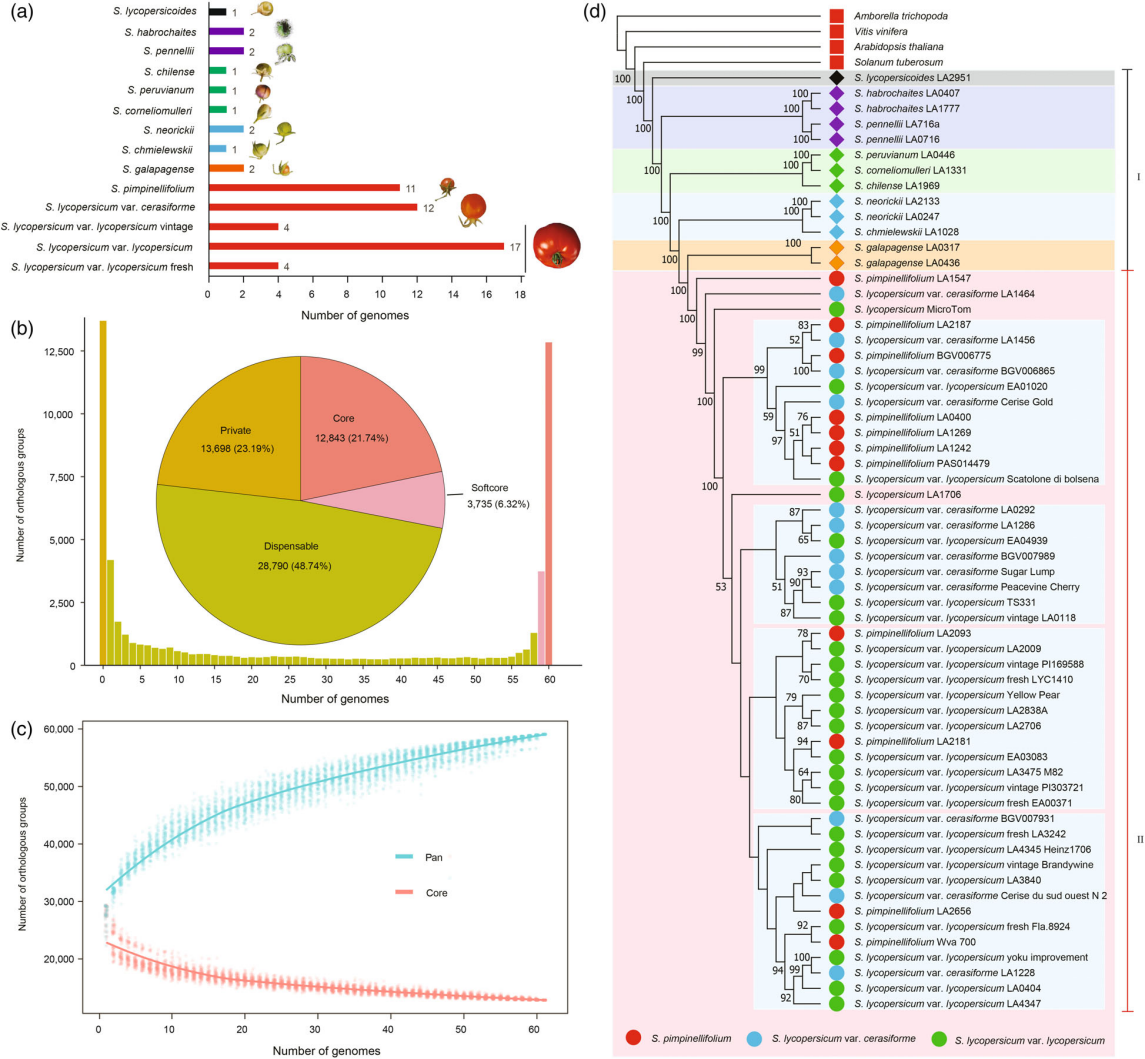
Statistics and phylogeny of 61 tomato genomes. (a) The number of tomato genomes representing each tomato species; (b) Composition of the tomato gene‐based super pan‐genome; (c) Simulations of the pan‐genome and core‐genome size in the tomato clade; (d) phylogeny of the 61 genomes in the tomato clade.

We collated 61 tomato genomic sequences, encompassing 13 distant wild relatives, 11 ancestral wild species (SP), 12 early domesticates (SLC), four early cultivars (vintage), 17 modern domesticates and four modern cultivars (fresh market), to construct the tomato gene‐based super‐pangenome (Figure 1a, Table S1). To eliminate annotation artefacts, the representative transcripts with transposable elements were filtered out to obtain clean functional protein‐coding gene sets that ranged from 31 056 (*S. peruvianum*, LA0446) to 38 584 (*S. neorickii*, LA2133). It is noteworthy that a total of 12 896 protein sequences in one tomato distant wild species, *S. pennellii* (LA716a), were identified as transposable element repeats, which were removed from the gene set (Figure S1). Finally, a total of 2 092 298 protein‐coding genes were used to construct the gene‐based tomato super‐pangenome. The resulting super‐pangenome contained 59 066 orthologous groups where 12 843 (21.7%) groups contained genes from all tomato genomes recognized as the core pan‐gene set. Moreover, 3735 (6.3%), 28 790 (48.7%), and 13 698 (23.2%) groups are classified as softcore (present in >99% of the 61 genomes), dispensable (present in 1–99% of the genomes), and private (present in <1% of the genomes) pan‐gene sets, respectively (Figure 1b). The private pan‐gene set includes 13 072 orthologous groups comprised of single‐copy genes and 626 orthologous groups with multiple‐copy genes in each of the 61 tomato individuals. Modelling of the tomato gene‐based super‐pangenome size suggested a closed or restricted pan‐genome, with a finite number of both pan and core pan‐genes (Figure 1c).

### New phylogeny of distant tomato wild relatives and cultivated tomatoes

Using four representative angiosperm species as the outgroup, a maximum‐likelihood phylogenetic tree of the 61 tomato genomes could separate them into two major groups (Figure 1d). In Group I, 13 distant wild relatives were clustered into five distinct subgroups, and *S. lycopersicoides* showed a much closer relationship to *Solanum tuberosum* than other distant tomato wild species, consistent with a previous report (Yu *et al*., 2022). The phylogeny of 65 genomes robustly supports the clustering of two *S. galapagense* individuals (LA0436 and LA0317) as being more ancestral than the wild species (SP). This relationship is supported by a 100% bootstrap value, which indicates a high level of confidence in this position within the phylogenetic tree. Our finding may rectify the long‐standing misunderstanding about the phylogeny of *S. galapagense* (Li *et al*., 2023; Yu *et al*., 2022). Eleven ancestral species, 12 early domesticates, four early cultivars, 17 modern domesticates and four modern cultivars were clustered into Group II. Interestingly, three tomato species: SP (LA1547), SLC (LA1464) and SLL (LA3911, Micro‐Tom) were more ancestral than the remaining tomatoes (Xue *et al*., 2023). The remaining 10 SPs, 11 early domesticates, four early cultivars, 16 modern domesticates and four modern cultivars were clustered and distributed into different subgroups of Group II without a clear evolutionary relationship from tomato ancestor to cultivar. Our results reveal the new phylogenetic relationships in tomatoes that are not fully in agreement with the current two‐phase domestication and improvement processes. Thus, we suggest that interspecies hybridization or genetic introgression could have occurred among the tomatoes of the phylogenetic tree in the gene‐based super‐pangenome (Gibson *et al*., 2021; Pease *et al*., 2016).

### Divergence of pan‐gene sets in the super‐pangenome

**Figure 2:**
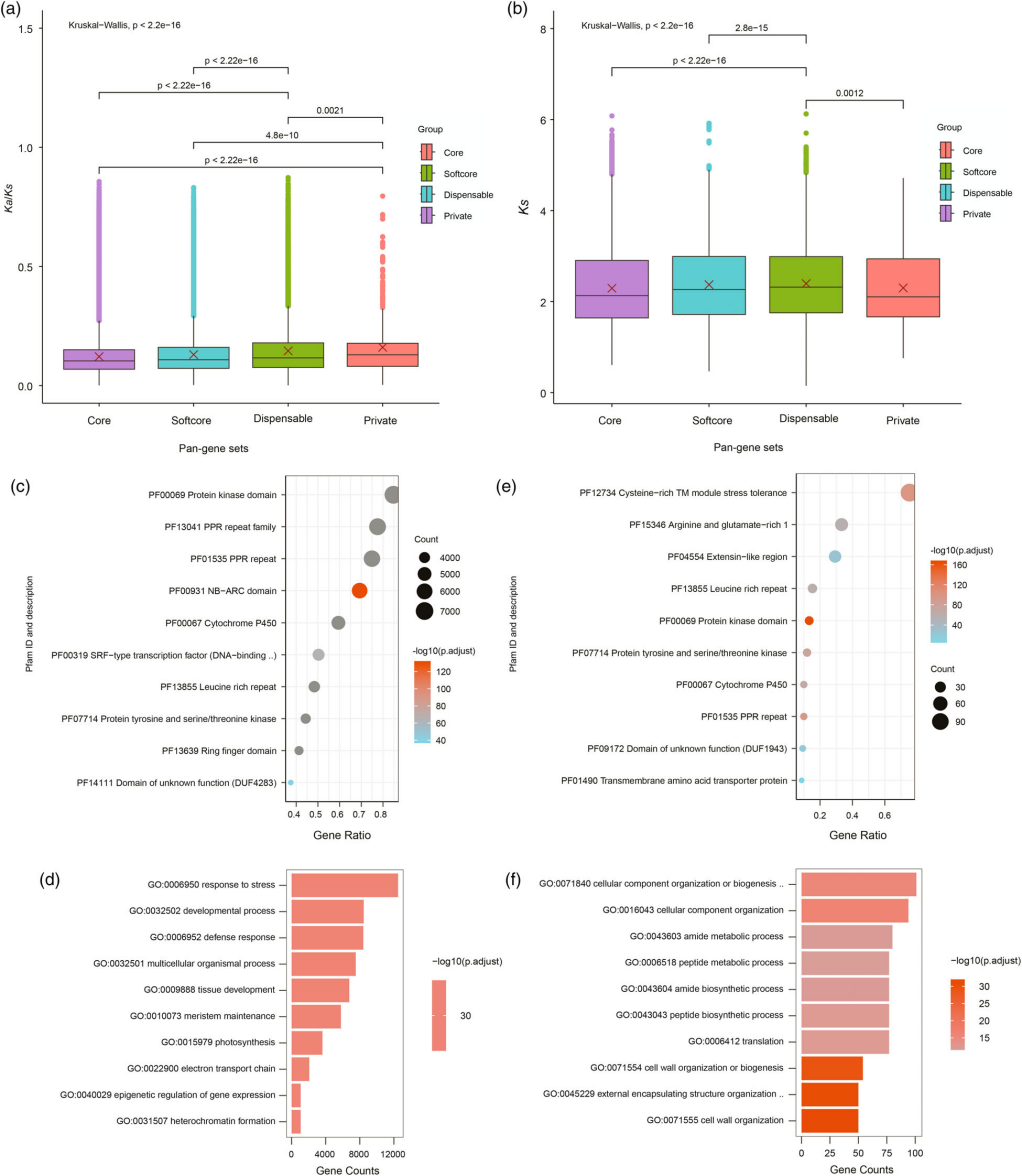
Evolution and divergence of the dispensable and private pan‐genes in the tomato gene‐based super pan‐genome. (a) *K*a/*K*s analyses statistics of core, softcore, dispensable and private pan‐genes; (b) *K*s analyses statistics of core, softcore, dispensable and private pan‐genes; (c and d) Pfam and GO (gene ontology) enrichment analysis of the dispensable pan‐genes; (e and f) Pfam and GO enrichment analysis of the private pan‐genes.

We next studied the ratios of the number of nonsynonymous substitutions per nonsynonymous site (*K*_a_) to the number of synonymous substitutions per synonymous site (*K*_s_) for the orthologous gene pairs between *Vitis vinifera* and the tomatoes. Most *K*_a_/*K*_s_ values were <1.0 (Fisher *P*‐value <0.05), indicating purifying or negative selection of these genes. The mean *K*_a_/*K*_s_ value of the orthologous gene pairs between the core pan‐gene set and *V. vinifera* genes was the lowest (0.1216; Fisher *P*‐value < 0.05) while that between the private pan‐gene set and *V. vinifera* genes was the highest (0.1606; Fisher *P*‐value ≤ 0.05), suggesting that the core pan‐genes have experienced a stronger selection pressure. However, there was no significant difference between the *K*_a_/*K*_s_ values of the orthologous gene pairs in dispensable and private pan‐gene sets compared to *V. vinifera* genes (Figure 2a). The mean *K*_s_ value of orthologous gene pairs between the core pan‐gene set and *V. vinifera* genes was the lowest (2.293; Fisher *P*‐value < 0.05). The results indicate that the highly conserved core pan‐genes had the lowest sequence divergence, which was the highest between the dispensable pan‐gene set and *V. vinifera* genes (2.3963; Fisher *P*‐value < 0.05). However, there was no significant difference between the *K*_s_ values of the orthologous gene pairs in dispensable and private pan‐gene sets compared to the *V. vinifera* genes (Figure 2b).

The enrichment analysis of protein families (Pfam) demonstrated that the dispensable pan‐genes are mainly enriched with the protein kinase, pentatricopeptide repeat (PPR), R proteins, Cytochrome P450, Serum response factor (SRF) and leucine‐rich repeat (LRR) (Figure 2c). These are mainly involved in biological processes related to stress responses, defence responses, developmental, multicellular organismal and tissue development (Figure 2d). The most enriched genes were related to stress tolerance, such as LRR‐RLK and NBS‐LRR genes in the dispensable pan‐gene set (Barragan and Weigel, 2021; Man *et al*., 2020). The private pan‐genes were found to be enriched in Cysteine‐rich TM module stress tolerance (CYSTM), arginine and glutamate‐rich 1 (ARGLU1), and extensin‐like region (Figure 2e). Interestingly, many private pan‐genes were enriched in the same gene families in the dispensable pan‐gene set, implicating that these gene super families have been strongly diversified to generate specific gene members of corresponding gene families in different tomato genomes. Genes from these families are mainly involved in the biological processes of cellular component organization or biogenesis, amide metabolic and biosynthetic, and peptide metabolic and biosynthetic (Figure 2f).

### Origin and evolution of duplicated tomato genes

**Figure 3:**
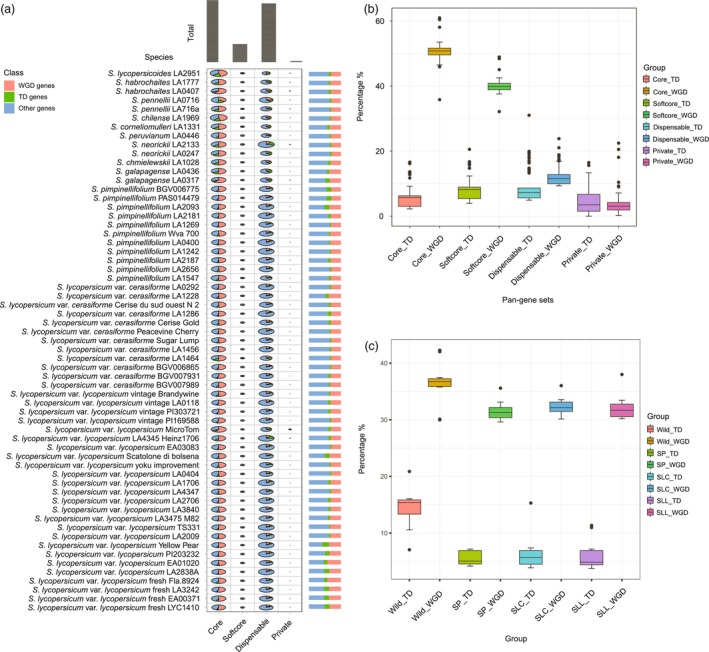
Comparisons of whole‐genome duplicated (WGD) and tandemly duplicated (TD) genes in tomato individuals, pan‐gene sets and tomato groups. (a) The distribution WGD and TD genes in tomato accessions; (b) Comparisons of WGD and TD genes in core, softcore, dispensable, and private pan‐genes; (c) Comparisons of the percentages of WGD and TD genes in four tomato groups. Other genes, genes without TD and WGD paralogs. Wild, wild distant relatives; SP, blueberry‐sized SP tomatoes; SLC, cherry‐sized SLC tomatoes; SLL, large‐fruited SLL tomatoes.

The tomato genome experienced a recent whole‐genome triplication (WGT) after the core‐eudicot WGT‐γ event (Sato *et al*., 2012). Since *V. vinifera* has not undergone any recent whole‐genome duplication (WGD) (Jaillon *et al*., 2007), *V. vinifera* genes were used to identify the duplicated genes in the tomato genomes. Tandem gene duplication (TD) is a mechanism for increasing the gene copy number in plant genomes (Qiao *et al*., 2019). Therefore, both WGT and TD may have contributed to the expansion of gene families to enhance biological functions. Each of the tomato genomes had >10 k genes from the WGD event, with *S. habrochaites* (LA0407) having the highest percentage (42.27%) among the 61 genomes (Figure 3a, Table S1). Twelve out of the 13 distant wild relatives had >10% of the total genes originating from the TD events, with *S. lycopersicoides* (LA2951) containing the highest (20.87%) percentage of genes. The early domesticated species, SLC (LA1464), SLL (LA4345, Heinz1706), and the dwarf cultivar of tomato (LA3911, Micro‐Tom) showed 15.3%, 11.34% and 10.97% genes with TD origin, respectively. The *S. peruvianum* (LA0446) genome only had 7.05% of the genes originating from the TD events. Interestingly, 46 out of 48 ancestral, cultivated and domesticated tomato genomes showed <8% of their total genes resulting from the TD events. Most of the TD genes were found to be enriched in the gene families of protein kinase, PPR repeats, Cytochrome P450, Leucine‐rich repeat, Ring finger and NB – ARC (Figure S1a), mainly involved in the response to stimulus, signalling, signal transduction, regulation of transcription by RNA polymerase II, transcription by RNA polymerase II and response to auxin (Figure S2b).

We further identified the influence of WGT and TD events on the four types of pan‐gene sets (Figure 3b, Table 1, Table S3). A decreasing trend from core to private was observed for the WGT event, indicating the presence of more WGT genes in the core pan‐gene set (50.68%). The mean percentage of TD genes in the softcore pan‐gene set (8.67%) was highest among the four pan‐gene sets (6.30% in core; 8.63% in dispensable; 5.96% in private) indicating the strongest influence of TD events on the softcore pan‐genes. The relaxed selection in the softcore gene set contained pan‐genes from 61 tomato genomes, which provided a larger contribution to the expansion or contraction of gene families than in the other pan‐gene sets. Furthermore, we identified the TD and WGD genes in the genomes of the distant wild species, blueberry‐sized SPs, cherry‐sized SLCs and large‐fruited SLLs (Figure 3c, Table S1). The tomato distant wild relative genomes were significantly more influenced by WGD and TD events than the rest of the tomato groups, and the SLCs experienced a stronger influence of both events than the SP and SLL tomatoes (Figure 3c).

**Table 1:** The whole‐genome and tandemly duplicated genes in the four pan‐gene sets

Gene set	Total genes	Whole‐genome duplicated (WGD) genes	Percentage (WGD/Total, %)	Tandemly duplicated (TD) genes	Percentage (TD/Total, %)
Core	927 650	470 128	50.68	58 403	6.30
Softcore	269 812	108 418	40.18	23 396	8.67
Dispensable	879 817	106 295	12.08	75 905	8.63
Private	15 019	618	4.11	895	5.96

### Evolution, domestication and improvement in tomato clade

**Figure 4:**
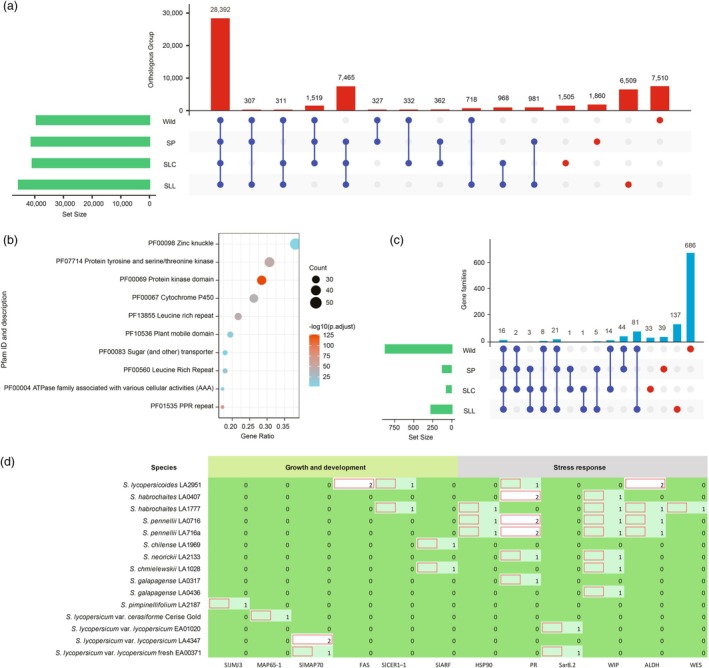
Genes or gene families that have been affected during evolution and domestication in the tomato clade. (a) Variation of orthologous groups in the four tomato taxa. Red solid circles represent specific orthologous groups; (b) Pfam enrichment analysis of specific genes in tomato distant wild relatives; (c) Variation of protein families in the four tomato groups. Red solid circles represent shared and specific protein families; (d) Genes related to growth and development, and stress responses in tomato individuals. Yellow rectangles represent the presence of target genes and red border rectangles represent the numbers of target genes in tomato individuals.

The two‐stage evolution of cultivated tomatoes provides an opportunity to investigate retained or lost genes during evolution, domestication and improvement from the distant wild relatives, blueberry‐sized SPs and cherry‐sized SLCs to the large‐fruited SLLs (Blanca *et al*., 2012, 2015). We evaluated the retained or lost genes in specific orthologous groups among the four tomato groups. There were 7510, 1860, 1505 and 6509 specific orthologous groups, harbouring 13 363, 2302, 1603 and 8030 genes in the distant wild relatives, SPs, SLCs and SLLs, respectively (Figure 4a). Analyses of enriched protein families revealed that the specific orthologous groups in tomato distant wild relatives account for more gene members of cytochrome P450 and protein kinase families to dispensable and private pan‐gene sets than other tomato groups (Figure 4b). Moreover, specific orthologous groups in SPs, SLCs and SLLs contributed more gene members of the CYSTM and ARGLU1 families to dispensable and private pan‐gene sets than distant wild relatives (Figures S3–S5).

Some gene members were found to be group‐specific in the tomato clade due to the divergence of super gene families, but they belong to the same gene families with shared Pfam ID. For example, we identified members of the six gene families: ATP‐binding cassette (ABC) transporter, legume lectin domain, C2 domain, ABC‐2 type transporter, protein tyrosine and serine/threonine kinase, and LRR in all four tomato groups, which indicated a large sequence diversity in these gene families in the tomato clade. Furthermore, we found 686 specific Pfam IDs in the distant wild relatives compared to 39, 33 and 137 in the SPs, SLCs, and SLLs, respectively (Figure 4c). Analyses of specific Pfam IDs resulted in the discovery of several novel genes related to fruit traits (e.g. fruit shape, ripening, and storability) as well as stress response (e.g. disease resistance and salt tolerance), which were found in one tomato group but were absent in others. We thus propose that these genes in the distant wild relatives potentially represent important functional genes for future tomato breeding, largely due to their loss during tomato evolution, domestication and improvement (Table S4).

### Presence/absence variation genes related to fruit trait and stress tolerance

Presence/absence variation (PAV) of genes can result in variable phenotypes or traits in tomato accessions (Gao *et al*., 2019). An important agronomic trait in modern tomato breeding programs is fruit size, which exhibits dramatic variation among the distant wild relatives and large‐fruited SLLs. For instance, we detected two tomato fruit size and locule number genes, *FASCIATED* (*FAS*) encoding a YABBY protein only in the distant wild relative, *S. lycopersicoides* (LA2951) (Cong *et al*., 2008; Slugina *et al*., 2020), three *SlMAP70* genes encoding microtubule‐associated protein 70 in the modern cultivar SLL fresh (EA00371) and modern domesticated SLL (LA4347) (Bao *et al*., 2024), and one *MAP65‐1* gene in early domesticated SLC (Cerise Gold) (Bao *et al*., 2024) (Figure 4d). These fruit size‐related genes may contribute to functional enhancement of morphological variation in tomato breeding. Moreover, we detected the fruit ripening‐related genes, *SlJMJ3* encoding a C5HC2 zinc finger protein in tomato ancestral species, *S. pimpinellifolium* (LA2187) (Li *et al*., 2024), the fruit postharvest shelf life‐related genes, *CER1–1* encoding a WAX2 C‐terminal domain containing protein in *S. habrochaites* (LA1777) and *S. lycopersicoides* (LA2951) (Wu *et al*., 2022), and the tomato fruit set gene, *ARF* encoding an auxin response factor in *S. chilense* (LA1969) and *S. chmielewskii* (LA1028) (Hu *et al*., 2023).

Stress defence genes are associated with response and tolerance to abiotic and biotic stresses (Adem *et al*., 2020; Shabala *et al*., 2020; Zhao *et al*., 2019). Here, we identified three viral infection‐inducible *Hsp90* genes that were only present in three accessions of distant wild relatives (*S. habrochaites*, LA1777; *S. pennellii*, LA0716 and LA716a) (Lubkowska *et al*., 2021), and nine *PR* genes of pathogenesis‐related protein Bet v 1 family in the distant wild relatives, *S. lycopersicoides* (LA2951), *S. habrochaites* (LA0407), *S. pennellii* (LA0716 and LA716a), *S. neorickii* (LA2133), *S. galapagense* (LA0317) (Wen *et al*., 1997). Interestingly, the identification of nine specific pathogenesis‐related genes in tomato distant wild relatives demonstrates that there was either an expansion of these gene families in these wild species or a loss in the cultivated accessions during domestication. Two *Sar8.2* genes were identified in two SLLs (SLL fresh, EA00371; SLL, LA4347) and these genes encoded *Sar8.2* family proteins that are induced by tobacco mosaic virus or salicylic acid (Alexander *et al*., 1992). Moreover, we identified genes conferring response to stress: one *WES* gene encoding wax ester synthase‐like Acyl‐CoA acyltransferase in a distant wild relative, *S. habrochaites* (LA1777) (Tomiyama *et al*., 2017), and five *ALDH* genes encoding aldehyde dehydrogenases that may respond to wounding stress via gene upregulation in distant wild relatives, *S. lycopersicoides* (LA2951), *S. habrochaites* (LA1777) and *S. pennellii* (LA0716 and LA716a) (Jimenez‐Lopez *et al*., 2016). We also identified seven *WIP* genes encoding wound‐induced proteins, conferring stress responses in seven distant wild relatives, *S. habrochaites* (LA0407 and LA1777), *S. pennellii* (LA0716 and LA716a), *S. neorickii* (LA2133), *S. chmielewskii* (LA1028) and *S. galapagense* (LA0436) (Zhou *et al*., 2022). The presence of these genes in the distant wild relatives suggests a potential strategy to improve fruit shelf life, fruit set and stress tolerance in elite tomato breeding.

### The LRR‐RLKome of leucine‐rich repeat receptor‐like kinases

**Figure 5:**
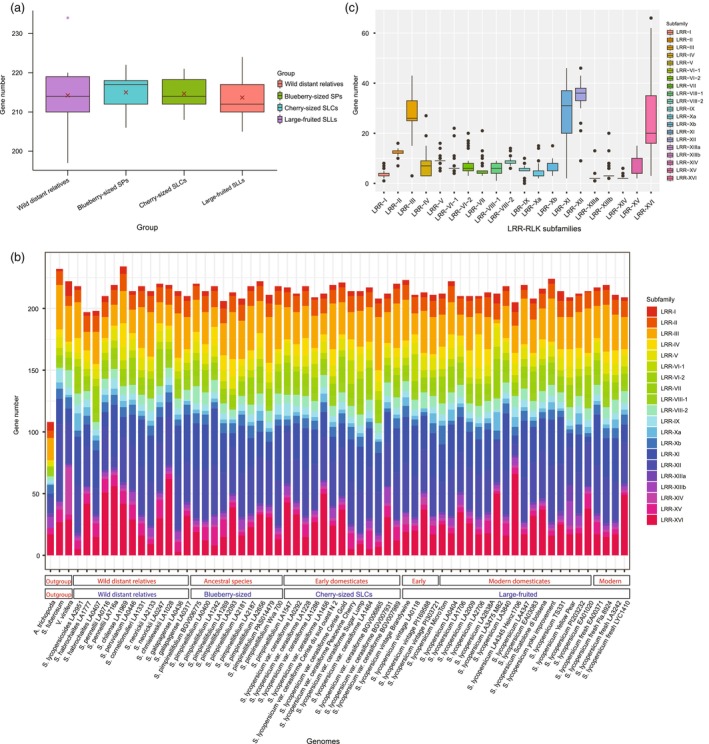
Statistics of LRR‐RLK genes in tomato taxa, gene subfamilies and tomato individuals. (a) Numbers of LRR‐RLK genes in the four tomato taxa; (b) Distribution of LRR‐RLK genes in subfamilies in different tomato individuals; (c) Comparison of LRR‐RLK genes in different LRR‐RLK gene subfamilies.

Members of the LRR‐RLK family play important roles in plant development and defence responses. They contain one or multiple extracellular LRR domains, a single‐pass TM, and an intracellular serine/threonine protein kinase domain (KD) (Man *et al*., 2020). In the current tomato gene‐based super‐pangenome, we constructed an LRR‐RLKome of 13 068 *LRR‐RLK* pan‐genes, representing LRR‐RLK genes across the tomato clade. The *LRR‐RLKs* account for an average of 0.6256% (214) of the total genes within each of the 61 tomato genomes, but with some variation between different genomes (Figure 5a). The members of the LRR‐RLK family were classified into 19 subfamilies (I‐XV), with LRR‐VI (LRR‐VI‐1 and LRR‐VI‐2), LRR‐VIII (LRR‐VIII‐1 and LRR‐VIII‐2), LRR‐X (LRR‐Xa and LRR‐Xb) and LRR‐XIII (LRR‐XIIIa and LRR‐XIIIb) having two separate subgroups that have previously been reported in plant genomes (Lehti‐Shiu *et al*., 2009). Importantly, we identified several LRR‐RLKs that do not belong to any of these subfamilies, which were placed in the ‘Unclassified’ category (called LRR‐XVI) as the 20th LRR‐RLK subfamily. When we classified the LRR‐RLK genes in individual accessions, we found a substantial diversity of gene subfamilies in different tomato groups (Figure 5b, Table S6). We then parsed all the LRR‐RLKs in the pan‐genome according to the 20 LRR‐RLK subfamilies and found that the LRR‐III, LRR‐XI, LRR‐XII and the new LRR‐XVI subfamilies contain higher numbers than the other ones (Figure 5c). Most of the LRR‐RLK genes (9705), representing 74.27% of the total LRR‐RLKs, constituted a core pan‐gene set, with 2317, 1029, and only two LRR‐RLK genes belonging to the softcore, dispensable and private pan‐gene sets of the tomato clade. Our results indicated that the LRR‐RLK genes in the tomato clade have relatively conserved evolution but have experienced strong selection pressure during domestication. There were 6571 (50.28%) LRR‐RLK genes that were generated during the WGT event, and 769 (5.88%) that were generated from TD events.

### Rapidly evolving and positively selected tomato genes

Using the branch model, we identified rapidly evolving genes with two types of evolutionary branches as foregrounds: (i) 52 rapidly evolving genes within all the tomato genomes (Table S6), and (ii) 51 rapidly evolving genes with the SPs, SLCs and SLLs (Table 2, Table S7). Functional annotation suggested that these genes are mainly involved in growth and development, stress responses and signal transduction. For the analysis in (i), several genes in the tomato clade were found to be evolving significantly faster. Some genes evolved more rapidly in the SPs, SLCs and SLLs accessions compared to the distant tomato relatives and outgroup species. For instance, *highly ABA‐induced* (*HAI3*) encodes one of the ABA coreceptors that modulate plant water use efficiency and drought tolerance (Bhaskara *et al*., 2012). *CYP711A1*/*MAX1* is a member of the Cytochrome P450 superfamily involved in strigolactone biosynthesis and heat and freezing tolerance. Some Cytochrome P450 are specific repressors of vegetative axillary bud formation by the axillary meristem (Sigalas *et al*., 2023; Wang *et al*., 2023). It was noteworthy that 14 genes were found to be evolving significantly faster than the species in the two types (i and ii) of analyses. For example, *WRKY9*, encoding WRKY DNA‐binding protein 9, is involved in increasing root suberin and salt tolerance (Krishnamurthy *et al*., 2021). *Cyclic nucleotide‐gated channel 15* (*CNGC15*) is mainly involved in the response to calcium transport and signalling (Tipper *et al*., 2023; Wang *et al*., 2021).

**Table 2:** Rapidly evolving and positively selected genes in the tomato clade

Categories	No. rapidly evolving genes	No. positively selected genes
Foreground: all tomatoes	52	2
Foreground: red‐fruited tomatoes	51	7

Using the branch‐site model, we detected two positively selected genes belonging to the *EMB2759* and *GRAS* gene families in the tomato clade with all the tomato genomes serving as the foreground (Table S8). *EMB2759*, encoding embryo defective 2759, may affect pathogen response and cell cycle during geminivirus infection (Ascencio‐Ibáñez *et al*., 2008), and GRAS family transcription factors are regulators of growth and development and multiple stresses (Waseem *et al*., 2022). We also detected seven positively selected genes when the distant relatives and outgroup representatives served as the background (Table S9). Of these, *CLB*, encoding a C2 calcium/lipid‐binding plant phosphoribosyltransferase family protein, is a repressor of abiotic stress responses (Xiao *et al*., 2022). *ABH* encodes an alpha/beta‐hydrolase superfamily protein and its structurally important fold works as the core structure of phytohormone and ligand receptors in the signalling pathways of gibberellin, strigolactone and Karrikin (Mindrebo *et al*., 2016).

### Comprehensive platform for tomato gene‐based super‐pangenome

**Figure 6:**
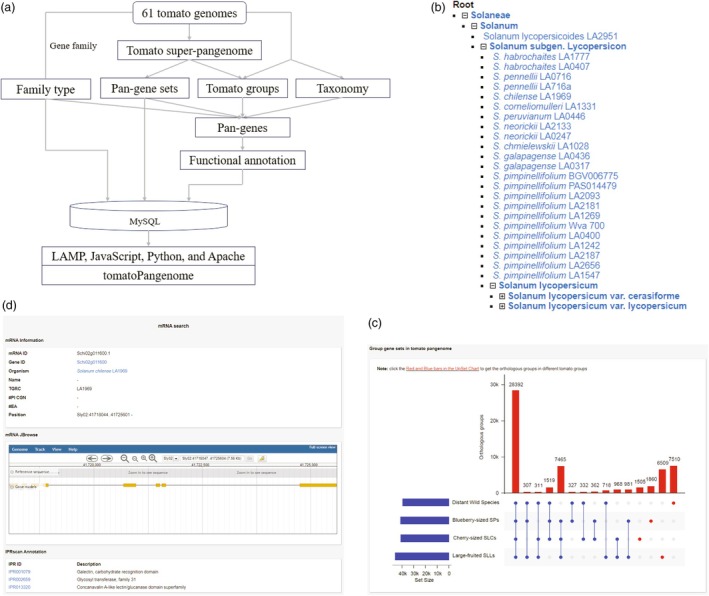
Workflow of tomatoPangenome development and screenshots of main functions in tomatoPangenome. (a) Workflow of tomatPangenome development; (b) Taxonomy of tomato species organized in tomatoPangenome; (c) The display of orthologous groups within tomato groups; (d) The module of basic information and functional annotations of pan‐genes.

To explore the genomic data of the tomato super‐pangenome, we have developed tomatoPangenome, a comprehensive platform designed to interrogate the tomato gene‐based super‐pangenome (Figure 6a). This user‐friendly database is accessible at http://www.varnatech.cn/tomatoPan/ and serves as a hub for exploring pan‐genes in tomato. Our platform provides a wealth of detailed information for each tomato genome, organized by taxonomy (Figure 6b) and genetic groups. The tomato super‐pangenome is structured around four distinct gene sets: core, softcore, dispensable, and private, and genetic groups (Figure 6c), while the gene family section focuses on LRR‐RLK genes within the tomato clade. For pan‐genes, tomatoPangenome delivers essential information and functional annotations, leveraging cross‐links to public databases (Figure 6d). Furthermore, tomatoPangenome is equipped with user‐friendly, customized tools such as BLAST, JBrowse and MISAweb, enhancing the utility of the platform for genomic research. Users can access downloadable types of pan‐gene data, facilitating research in tomato genomics, breeding and the identification of genomic components for genome‐assisted breeding.

## Discussion

In this study, we constructed a gene‐based super‐pangenome for tomato, integrating 59 066 orthologous groups from 61 diverse tomato genomes that were integrated into a new tomatoPangenome platform. Our analysis increased the total number of orthologous groups by 35.34% compared to the previously documented 40 457 (Li *et al*., 2023). This overall increase is juxtaposed with a substantial reduction in the core orthologous groups, now totalling 13 932, compared to the previously reported 23 839 (Li *et al*., 2023). Moreover, the proportion of private pan‐genes, accounting for 23.2% of the total, surpasses the earlier documented 7.6% of accession‐specific pan‐genes (Gao *et al*., 2019). This expanded super‐pangenome offers an enriched tapestry of genetic diversity, presenting a valuable genetic resource to the tomato research community. Our analysis underscores the contribution of TD events to the expansion of gene families, particularly in tomato distant wild relatives. Furthermore, we identified specific orthologous groups across various tomato groups, which exhibit variations in gene content, indicative of distinct evolutionary pressures. Genes associated with key fruit traits and stress tolerance display PAV among different tomato accessions, presenting potential targets for crop improvement. The in‐depth analysis of the LRR‐RLK gene family revealed significant sequence diversity and conservation across tomato genomes. Particularly, only two genes in the tomato clade exhibited positive selection compared to the outgroup species. In our analysis, we employed 1249 gene pairs of single‐copy orthologous genes to ensure precise and conserved comparisons between the outgroup species and tomatoes. We surmised that while single‐copy orthologous genes are generally highly conserved across these species, a small number of them, experienced strong selection pressures from the environment, would display positive selection, thereby facilitating adaptation to changing environments. Additionally, we identified genes such as *HAI3*, *CYP711A1/MAX1*, *WRKY9*, *CNGC15* and *Hsp90* undergoing rapid evolution and positive selection, involved in processes such as growth, development, stress responses and signal transduction. The collective identification of these genes, particularly those linked to fruit traits, stress tolerance and disease resistance, opens avenues for enhancing elite tomato cultivars through future breeding programs.

Another highlight of this study is a new evolutionary placement of wild species *S. galapagense* in the phylogenetic tree of the genus *Solanum*. Spooner *et al*. (2005) reported that *S. cheesmaniae* and *S. galapagense* originated from the Galápagos Islands, grouped together, but close to the group of SPs and SLLs, via the AFLPs analysis for phylogeny in wild tomatoes. Morphological examination of fruit colour characteristics indicates that *S. cheesmaniae* and *S. galapagense* typically exhibit yellow and orange fruit pigmentation, whereas SPs and SLLs predominantly display red fruit coloration, but they all belonged to a well‐supported clade (100% bootstrap) (Spooner *et al*., 2005). It was reported that *S. cheesmaniae* and *S. galapagense* distributed between the SPs and SLLs groups in the SNP‐based phylogenetic trees for distinct collections of tomato accessions with different versions (SL2.40, SL3.0 and SL4.0) of the tomato reference genome (Alonge *et al*., 2020; Gao *et al*., 2019; Gibson *et al*., 2021; Lin *et al*., 2014). SNP‐based phylogenetic tree analyses can reflect variations across the entire single linear reference genome (SLL), including non‐coding genes. However, the tomato distant wild species and SPs encoded significantly more genes than SLLs (Gao *et al*., 2019). Therefore, these SNP‐based phylogenetic trees are not likely to supply a concise species evolutionary relationship in the genus *Solanum*. In contrast, highly conserved protein sequences are more likely to reflect more distant evolutionary relationships due to functional constraints that result in fewer sequence changes. Gao *et al*. (2019) and Li *et al*. (2023) employed the protein sequences of single‐copy orthologous groups among tomato wild and cultivated species, and outgroup species to infer the phylogeny of the wild species *S. galapagense* distributing between SPs and SLLs, but all the outgroup species belonged to the genus *Solanum* (Gao *et al*., 2019; Li *et al*., 2023). Specific functional genes on certain chromosomes show genetic affinities with wild tomato lineages, suggesting shared ancestry or adaptive traits, while others indicate distinct evolutionary interactions or selective pressures, potentially due to gene flow, introgression, or adaptation to similar environmental challenges, with more ancient genomes beyond the *Solanum* genus highlighting distant evolutionary relationships. The identification of such ancient genomic elements in the tomato lineage provides insights into the deep evolutionary history and the basal genetic architecture of these species. Here, the large number (61) of tomato genomes and the selection of four key species as outgroups have enabled a precise phylogenomic analysis. *Amborella*, which is recognized as the most basal extant flowering plant (DePamphilis *et al*., 2013), grape, a true diploid without a recent genome duplication after the angiosperm WGT‐γ event (Jaillon *et al*., 2007), the model species Arabidopsis (Initiative, 2000) and *S*. *tuberosum*, a close relative to tomato in the *Solanum* genus (Boris *et al*., 2011) were recognized as outgroup species of the phylogenetic tree. In contrast to the number of single‐copy orthologous genes derived from the genomes of the genus *Solanum* as reported in the study by Li *et al*. (2023), the incorporation of three outgroup species may lead to a reduction in the number of single‐copy orthologous genes. However, the identified single‐copy orthologous genes originate exclusively from the ancestral orthologous genes shared among the common ancestors of these three outgroup species and the 61 tomato species. This remarkable conservation of ancestral orthologous genes serves as a precise and reliable indicator of the phylogenetic relationships between the three outgroup species and tomatoes. As a result, these four species and almost all genome‐sequenced with the optimal protein sequence‐based phylogenetic tree inferred a new evolutionary position of *S. galapagense* in the tomato clade.

Genes that differed between the four tomato groups (distant wild species, blueberry‐sized SPs, cherry‐sized SLCs and large‐fruited SLLs) were investigated, representing tomato evolution, domestication and improvement. We observed that in some cases, members of super gene families were distributed to more than two tomato genomes, while in other cases members exist in specific orthologous groups, indicating divergence of gene families is related to key traits for fruit size and locule number, shelf life and stress resistance in certain tomato accessions. For example, the *FAS* gene family with 546 genes that in the tomato clade has experienced not only sequence divergence but also expansion and contraction to potentially regulate the diversity of tomato fruit size and locule numbers in different accessions (Table S10). In addition, several agronomically important genes such as a gene associated with fruit storage (*CER1‐1*), shelf life (*ARF*), disease resistance (*Hsp90* and *PR*) and stress responses (*WIP*, *ALDH* and *WES*) were identified in distant wild relatives that were absent from the other three tomato groups (Table S4). The identification of genes related to fruit traits, stress tolerance and disease resistance suggests opportunities for improving elite tomato cultivars through breeding because these genes have been lost during tomato domestication and breeding. This study provides a genetic repertoire in the tomato clade and is geared toward accelerating the utilization of functional genes in genome‐assisted breeding.

In conclusion, the current tomato gene‐based super‐pangenome offers valuable genetic resources or gene repertoire for the research community and breeding programs with a user‐friendly database available at http://www.varnatech.cn/tomatoPan/ (Figure S5). The identification of genes associated with key fruit traits and stress tolerance as well as gene family expansions and evolutionary adaptations provides a foundation for enhancing elite tomato cultivars through strategic breeding. This study will contribute significantly to the sustainable production of high‐quality, climate‐resilient tomatoes to meet future food demands.

## Materials and methods

### Data sources

The genomic data, including assembled genome sequences, coding sequences, predicted protein sequences and gene location files of eight tomato relatives were downloaded from http://caastomato.biocloud.net/home (Li *et al*., 2023). The *S. pennellii* (LA716a) genomic data were downloaded from https://solgenomics.net/organism/Solanum_pennellii/genome (Bolger *et al*., 2014). Two tomato wild distant relative genomic data (*S. habrochaites*, LA0407 and *S. galapagense*, LA0317) were downloaded from https://ngdc.cncb.ac.cn with the bioproject number: PRJCA008297 (Yu *et al*., 2022) and tomato model cultivar (MicroTom) genomic data was downloaded from MicroTomBase (https://eplant.njau.edu.cn/microTomBase/) (Xue *et al*., 2023). The genomic datasets of two wild tomato relatives, *S. neorickii* (LA2133) and *S. pennellii* (LA0716), which were assembled from PacBio HiFi data, were obtained from Dr. Zhangjun Fei's group and are available at http://ted.bti.cornell.edu/ftp/tomato_genome/. Sequences from 11 ancestral species (*S. pimpinellifolium*), 12 early domesticates (*S. lycopersicum* var. *cerasiforme*), 4 early cultivars (*S. lycopersicum* var. *lycopersicum* vintage), 16 modern domesticates (*S. lycopersicum* var. *lycopersicum*) and 4 modern cultivars (*S. lycopersicum* var. *lycopersicum* fresh) were downloaded from http://solomics.agis.org.cn/tomato/ (Zhou *et al*., 2022) and https://solgenomics.net/ftp/genomes/tomato100/. The genomic data from *A. trichopoda* (AMTR1.0.57), *V. vinifera* (v2.1), *A. thaliana* (Araport11) and *S. tuberosum* (PGSC_DM_v4.03) were downloaded from: https://plants.ensembl.org (Bolser *et al*., 2016), https://phytozome‐next.jgi.doe.gov/ (Jaillon *et al*., 2007), http://www.arabidopsis.org (Cheng *et al*., 2017) and http://spuddb.uga.edu/ (Sharma *et al*., 2013; Xu *et al*., 2011), respectively.

All the gene sets with evidence of alternative splicing were parsed and the longest transcripts served as the representative genes in different genomes. The transposon elements were filtered from the entire protein sequences using TEsorter v1.4.6 with the parameters ‘‐st prot ‐p 160 ‐cov 30 ‐eval 1e‐5 ‐dp2’ (Xue *et al*., 2023). Then, transposon elements were further filtered with keyword ‘transposon’ in the InterProScan v5.52‐86.0 annotation (Paysan‐Lafosse *et al*., 2023). Finally, a total of 2 092 298 clean functional tomato protein‐coding genes in 61 tomato genomes were used for downstream analyses. All filtered tomato protein sequences from individual genomes were evaluated with Benchmarking Universal Single‐Copy Orthologs (BUSCO) version 5.4.7 (Berkeley *et al*., 2021), and if the BUSCO values were >90%, the tomato accessions were retained for subsequent analyses. For duplicate tomato accessions, the accessions with higher BUSCO values were retained. In total, 61 tomato genomes were used in this analysis.

### Construction of gene‐based super‐pangenome

OrthoFinder version 2.5.4 was employed to identify orthologous groups with the protein sequences from 61 tomato genomes, using default parameters (Emms and Kelly, 2019). The output files of orthologous groups including ‘Orthogroups.tsv’ and ‘Orthogroups_UnassignedGenes.tsv’ were merged into one file as the final data set comprising tomato orthologous groups for downstream analyses. The orthologous groups that recognized all genomes were the core gene set, and the rest of the orthologous groups, present in >99%, 1–99%, and <1% of the samples present were defined as softcore, dispensable and private gene sets, respectively.

### Phylogeny of tomato accessions

A total of 1249 single‐copy orthologs from 61 genomes of tomato wild distant species, ancestral, cultivated and domesticated tomato and four representative plant genomes including *A. trichopoda*, *V. vinifera, A. thaliana*, and *S. tuberosum*—a close relative to tomato in the *Solanum* genus (Boris *et al*., 2011), were used in a phylogenetic analysis. Multiple sequence alignments of single‐copy orthologous sequences were implemented with MAFFT v7.520 (Rozewicki *et al*., 2019), and the conserved sequences were retrieved from aligned sequences using GBLOCKS 0.91b, with the parameters: ‘‐b4=5 ‐b5=h ‐t=p ‐e=.2’ (Castresana, 2000). The conserved sequences from the same sample were then merged into one sequence using SeqKit v0.3.1.1, with the parameter: ‘‐w’ (Shen *et al*., 2016). IQ‐Tree v2.3.1 with the parameters ‘‐m MFP ‐bb 1000 ‐bnni ‐T 30’ was used to perform a phylogenetic analysis that included four outgroup genomes and 58 tomato samples (Nguyen *et al*., 2015).

### Retrieval of whole‐genome and tandemly duplicated genes

The intermediate files from BLAST search results within each tomato genome or between two tomato genomes from OrthoFinder analyses were used as input files for the identification of the whole‐genome duplicated and tandemly duplicated genes. The BLAST results of orthologous gene pairs between tomato and grape genomes from OrthoFinder served as the input files for the syntenic analysis, and the MCscanX (11.13.2012) software was used to identify tomato whole‐genome duplicated genes within the syntenic regions between tomato and grape genomes (e = 1e^−20^, u = 1 and s = 15) (Wang *et al*., 2012). The BLAST results show paralogous gene pairs within individual tomato genomes from OrthoFinder as the input files for the tandem gene duplication analysis, and the adjacent paralogous genes within the same genomic regions were recognized as tandemly duplicated genes.

### Analysis of core, softcore, dispensable and private pan‐gene sets for *K*a, *K*s and *K*a/*K*s

The whole‐genome duplicated gene pairs between the tomato and grape genomes were analysed for rates of nonsynonymous (*K*a) and synonymous (*K*s) substitutions, as well as the *K*a/*K*s ratio, using the KaKs_Calculator2.0 software (Wang *et al*., 2010). This analysis independently generated 58 output files for *K*a, *K*s and *K*a/*K*s, respectively. The files containing *K*a values were consolidated into a single dataset. This dataset was then categorized into core, softcore, dispensable and private pan‐gene sets of *K*a values based on the classification of tomato genes within the whole‐genome gene pairs shared between the tomato and grape genomes. Subsequently, *K*s and *K*a/*K*s analyses were conducted on these categorized sets, adhering to the pipeline used for the *K*s analysis.

### Protein family and gene ontology enrichment analysis

The predicted protein sequences for the 61 genomes in the tomato clade were annotated using InterProScan v5.52‐86.0 with the parameters: ‘‐cpu 64 ‐goterms ‐dp ‐f tsv’ (Paysan‐Lafosse *et al*., 2023). Pfam ID and GO terms with their corresponding descriptions for each tomato gene were extracted from the InterProScan output files. Further, Pfam and GO annotation databases of core, softcore, dispensable and private pan‐gene sets, whole‐genome duplicated and tandemly duplicated gene sets, and individual tomato whole‐genome gene sets were all built to be used in different Pfam and GO enrichment analyses. Pfam enrichment analyses were conducted utilizing custom in‐house pipeline. Initially, a curated list of target genes, complete with Pfam IDs, was extracted from the established database. The compiled list was utilized to establish a mapping between Pfam IDs and their respective gene counts. For each Pfam ID, a set of numerical values was defined to represent the foreground: this included the count of genes annotated with the target Pfam ID from the curated gene list and the overall gene count within that list. Concurrently, the background was defined by the count of genes annotated with the target Pfam ID in the established database and the total gene count encompassed by the database. With these delineated foreground and background datasets, a Fisher's exact test was conducted to statistically assess the enrichment of each Pfam ID within the established database. The Fisher test adjusted *P*‐value < 0.05 was used as the threshold to obtain significantly enriched Pfam IDs and descriptions. Gene Ontology enrichment analyses were performed with the clusterProfiler v.4.2 R package (Wu *et al*., 2021), with a Fisher test adjusted *P*‐value of <0.05 as the threshold for significance.

### Presence/absence variation of genes or gene families during tomato evolution, domestication and improvement

Analyses of presence/absence variation (PAV) of genes classified tomato pan‐genes of orthologous groups into core, softcore, dispensable and private pan‐gene sets. To elucidate the genetic variation throughout the evolutionary, domestication and improvement processes of tomatoes, orthologous groups were categorized into distinct sets based on the taxonomic affiliation of tomato samples. This classification encompassed samples from diverse tomato groups, including wild distant relatives, blueberry‐sized SPs, cherry‐sized SLCs and large‐fruited SLLs. Orthologous groups assigned to the core pan‐gene set were excluded from the analysis, while those affiliated with the softcore, dispensable and private pan‐gene sets were retained. This selection was made to focus the investigation on the PAV of genes and gene families throughout the evolutionary, domestication and improvement trajectories of tomato species. Subsequently, the downstream analyses primarily concentrated on the orthologous groups that were specific to tomato groups.

The presence of specific genes with identical Pfam IDs across the four taxa suggests that they are members of the same gene families. Prior research has demonstrated that plant super gene families exhibit substantial sequence divergence, leading to the formation of distinct subfamilies, such as those observed in the Cytochrome P450 and UDP‐Glycosyltransferase (UGT) gene superfamilies (Xu *et al*., 2015; Yu *et al*., 2017). Consequently, members of super gene families with the same Pfam IDs might be distributed across different orthologous groups due to significant sequence divergence. Additionally, we scrutinized the specific Pfam IDs within the four tomato groups and proceeded to analyse PAV of agronomy‐related genes among tomato groups and accessions.

### Identification of LRR‐RLKs in the tomato clade

Based on the conserved domains or motifs of LRR‐RLKs, the Hidden Markov model (HMM) profiles of protein kinase domain (PF00069), LRR_1 (PF00560), LRR_2 (PF07723), LRR_3 (PF07725), LRR_4 (PF12799), LRR_5 (PF13306), LRR_6 (PF13516) and LRR_8 (PF13855) were retrieved from Pfam 36.0 (http://pfam‐legacy.xfam.org/) (Mistry *et al*., 2021). First, candidate protein kinases were identified among the 61 tomato genomes with HMM profile PF00069 using the HMMER v3.4 software, with ‘trusted cutoff’ as a threshold (http://hmmer.org/). High‐quality protein sequences of candidate protein kinases in each tomato accession were aligned with MAFFT v7.520 (Rozewicki *et al*., 2019) and the multiple alignment sequences were then used to construct tomato accession‐specific protein kinase profiles using the ‘hmmbuild’ module in the HMMER v3.4 software. With the rebuilt HMM profiles, the final protein kinases in each tomato genome were confirmed using the HMMER v3.4 software. Secondly, the final protein kinases in each tomato genome with extracellular LRR domains (LRR_1, LRR_2, LRR_3, LRR_4, LRR_5, LRR_6 and LRR_8) were identified using HMMER v3.4 software with the LRR HMM profile. The unique protein sequences were kept in each tomato genome. Thirdly, TMHMM v2.0 (https://services.healthtech.dtu.dk/services/TMHMM‐2.0/) (Krogh *et al*., 2001) was employed to identify the TM domains in the final protein kinases with LRRs in each tomato genome and the target protein sequences without TM domains were excluded. Finally, the protein kinases with LRR and TM domains from the 61 tomato genomes were classified as LRR‐RLK genes, which collectively constitute the LRR‐RLKome within the pangenome. The 19 released Arabidopsis members of the LRR‐RLK gene subfamilies (Lehti‐Shiu *et al*., 2009) were aligned to the LRR‐RLK genes from each tomato genome and the alignments used to construct phylogenetic trees using IQ‐Tree v2.3.1, with the parameters ‘‐m MFP ‐bb 1000 ‐bnni ‐T 30’ (Nguyen *et al*., 2015). The tomato LRR‐RLK genes clustering with the Arabidopsis representatives were recognized as the members of the different LRR‐RLK gene subfamilies based on the Arabidopsis gene representatives.

### Positively selected and rapidly evolving genes

A total of 1249 single‐copy orthologs from 61 tomato genomes and a phylogenetic tree constructed from 65 genomes were used to identify the positively selected and rapidly evolving genes in the tomato clade. MAFFT v7.520 was used to perform multiple sequence alignments of coding sequences of orthologous gene pairs (Rozewicki *et al*., 2019) and PAML v4.4b was used to estimate the dN/dS ratios with the multiple coding sequence alignments (Yang, 2007). Firstly, we estimated the dN/dS ratio values using branch models (mode = 2 and NSsite = 0) involving 65 genomes with the following parameters: Codonfreq = 2; kappa = 2.5; initial omega = 0.2. Three hypotheses were examined: (i) H0 hypothesis, where all branches have identical dN/dS ratio values; (ii) H1 hypothesis, where the branch of target tomato groups has a single dN/dS ratio value while the other branches have other identical dN/dS ratio values; and (iii) H2 hypothesis, where all branches have different dN/dS ratio values. An LRT (likelihood‐ratio test) was used to select target genes whose likelihood values of H1 were significantly larger (adjusted LRT *P*‐value of <0.01) than those of H0, and likelihood values of H2 were not significantly larger than those of H1. The genes with larger dN/dS ratio values in target tomato groups than in other branches were defined as rapidly evolving [rate (FDR)‐corrected *P*‐values (<0.01)]. The branch‐site models (model = 2 and NSsite = 2) were employed to identify the genes with positively selected sites in the tomato groups. The parameters of ‘fix_omega’ = 1 and ‘omega’ = 1 were used as a null hypothesis, but ‘fix_omega’ = 0 and ‘omega’ = 1.5 were used for the alternative hypothesis with the inferred phylogenetic tree. We used an FDR‐corrected LRT with *P*‐value (adjusted LRT *P*‐value) cutoff <0.01 to identify genes with positively selected sites in target tomato groups.

## Author contributions

JY and SX conceived this project. JY analysed the data and prepared the initial manuscript. QC, LY, MH, PZ, GC and XT performed data collection and analysis. SF fixed the code for drawing figures. ZHC and DE revised the manuscript. All authors read and approved the final manuscript.

## Funding

This work was supported by the Key Research and Development Program of Zhejiang (2024SSYS0099), and the Project of Xianghu Laboratory with Grant No. 2023C1S01002.

## Conflict of interest

None declared.

## Supporting information


**Figure S1** Statistics of cleaned and filtered protein‐coding genes in 61 tomato genomes.
**Figure S2** The Pfam and GO enrichment analysis of the entire TD genes in 61 tomato genomes. (a) The Pfam enrichment analysis; (b) The GO enrichment analysis.
**Figure S3** Protein family enrichment analysis of specific genes in the blueberry‐sized SPs.
**Figure S4** Protein family enrichment analysis of specific genes in the cherry‐sized SLCs.
**Figure S5** Protein family enrichment analysis of specific genes in the large‐fruited SLLs.
**Figure S6** Homepage of tomatoPangenome platform.

**Table S1** list of 61 tomato genomes.
**Table S2** Statistics of whole‐genome and tandemly duplicated genes within tomato accessions.
**Table S3** Statistics of whole‐genome and tandemly duplicated genes in core, softcore, dispensble, and private pan‐genes among different tomato accessions.
**Table S4** PAVs of the genes related to growth and development, and stress response.
**Table S5** Statistics of LRR‐RLK genes of different subfamilies in tomato accessions.
**Table S6** The rapidly evolving genes with the entire tomato genomes serving as the foreground.
**Table S7** The rapidly evolving genes with blueberry‐sized SPs, cherry‐sized SLCs, large‐fruited SLLs and one wild tomato, *S. galapagense*, serving as the foreground.
**Table S8** The positively selected genes in tomato clade with the entire tomato genomes serving as the foreground.
**Table S9** The positively selected genes with tomato distant relatives and outgroup representatives serving as the foreground.
**Table S10** Gene list of FAS genes in tomato accessions.

## Data Availability

Data sharing is not applicable to this article as no new data were created or analyzed in this study.
