# Supplementary material for: Phylogenomic and super‐pangenome analyses unveil the genetic landscape of tomato evolution and domestication
Source: Plant Biotechnol J. 2025 Jun 15;23(9):3783–97. doi: 10.1111/pbi.70199 (PMC12392952; doi:10.1111/pbi.70199)
Supplement: Supplementary file 1 — Figure S1 Statistics of cleaned and filtered protein‐coding genes in 61 tomato genomes. Figure S2 The Pfam and GO enrichment analysis of the entire TD genes in 61 tomato genomes. (a) The Pfam enrichment analysis; (b) The GO enrichment analysis. Figure S3 Protein family enrichment analysis of specific genes in the blueberry‐sized SPs. Figure S4 Protein family enrichment analysis of specific genes in the cherry‐sized SLCs. Figure S5 Protein family enrichment analysis of specific genes in the large‐fruited SLLs. Figure S6 Homepage of tomatoPangenome platform. [file PBI-23-3783-s002.docx]

**Supplemental data**


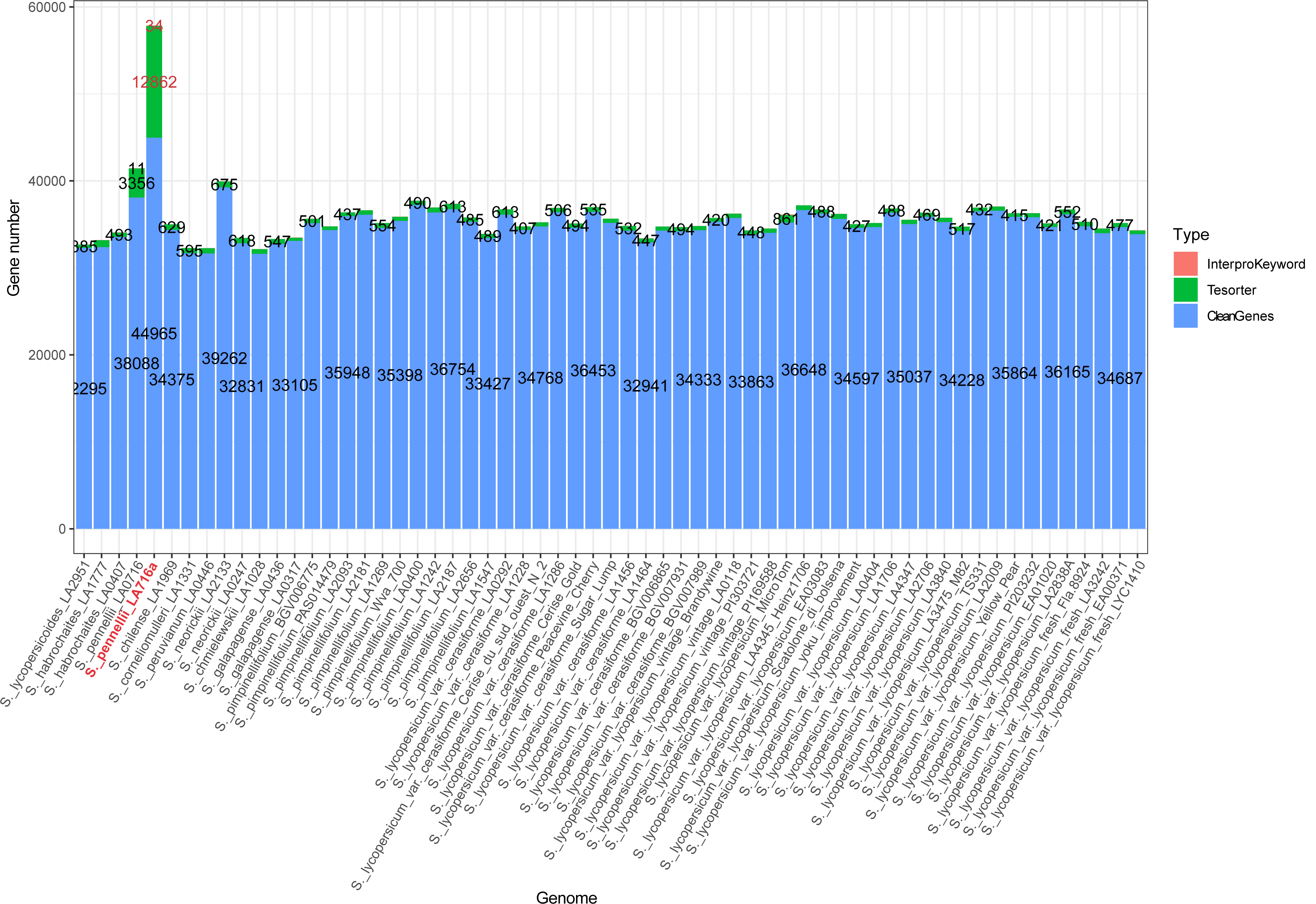


**Supplemental figure S1.** Statistics of cleaned and filtered protein-coding genes in 61 tomato genomes.


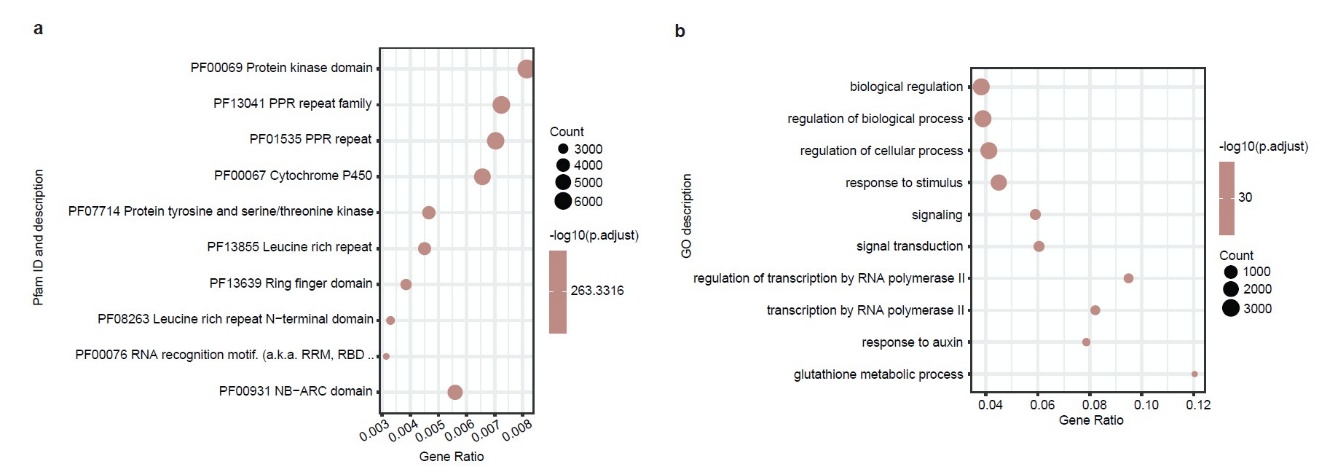


**Supplemental figure S2.** The Pfam and GO enrichment analysis of the entire TD genes in 61 tomato genomes. a. the Pfam enrichment analysis; b. the GO enrichment analysis.


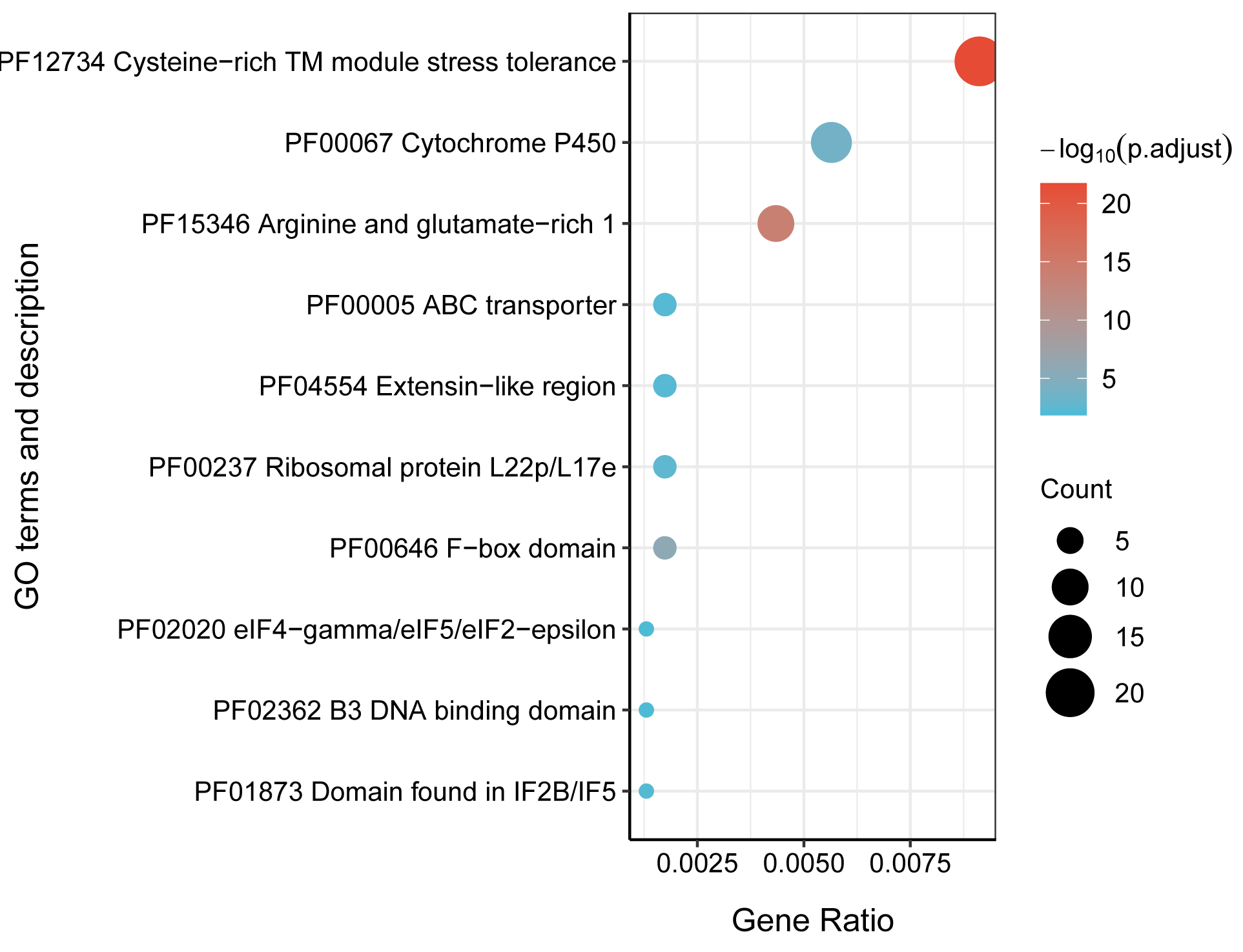


**Supplemental figure S3.** Protein family enrichment analysis of specific genes in the blueberry-sized SPs.


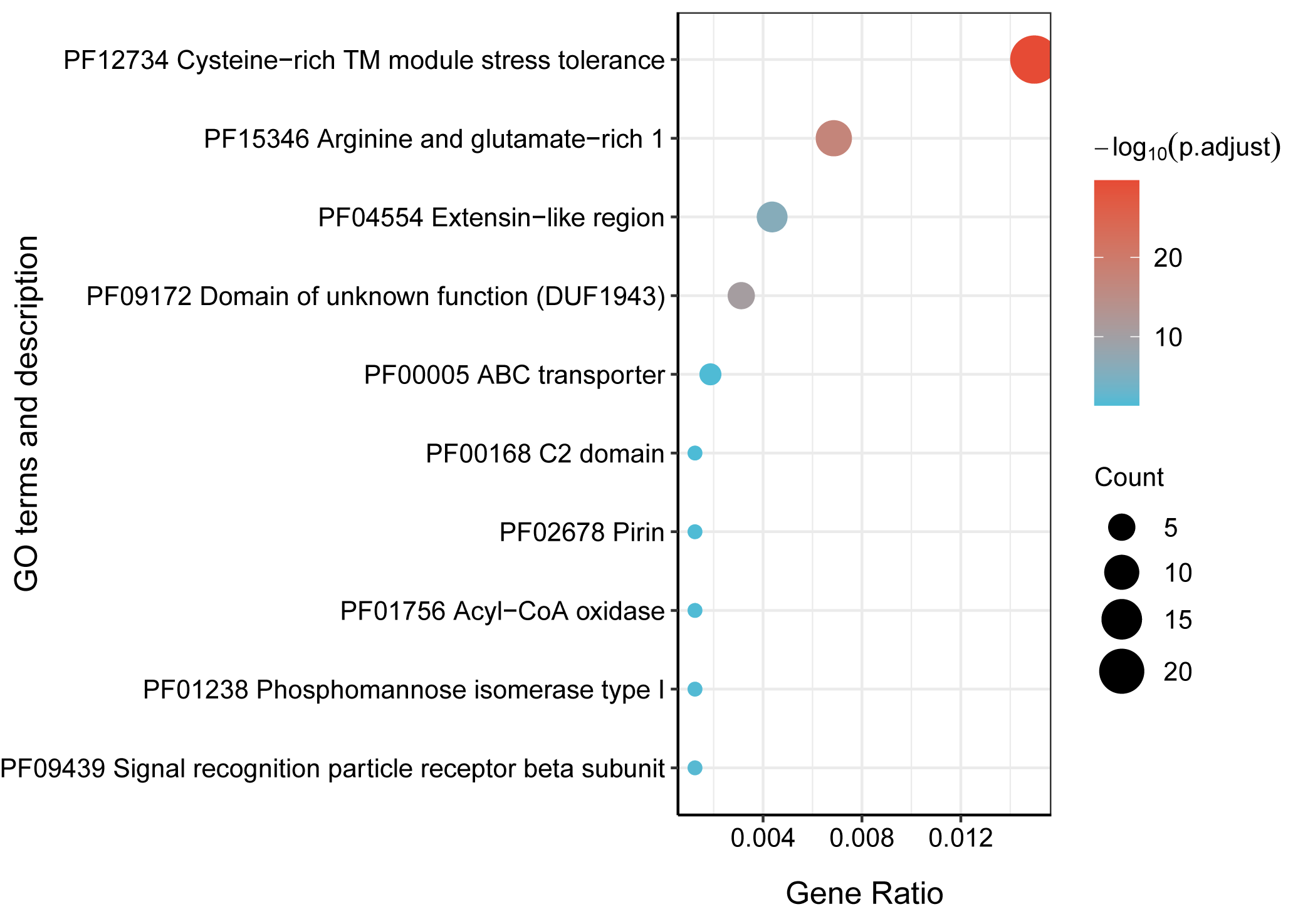


**Supplemental figure S4.** Protein family enrichment analysis of specific genes in the cherry-sized SLCs.


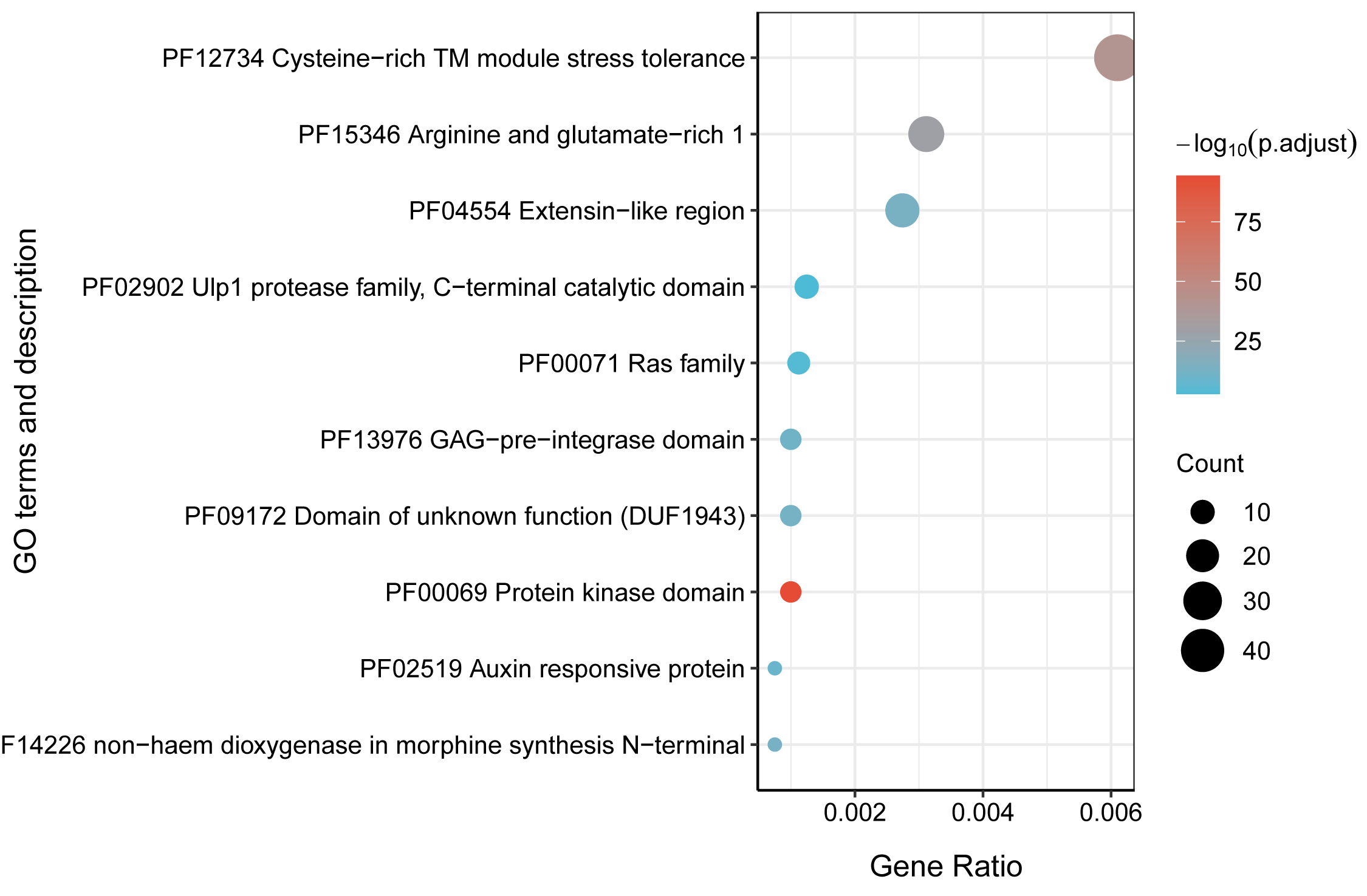


**Supplemental figure S5.** Protein family enrichment analysis of specific genes in the large-fruited SLLs.


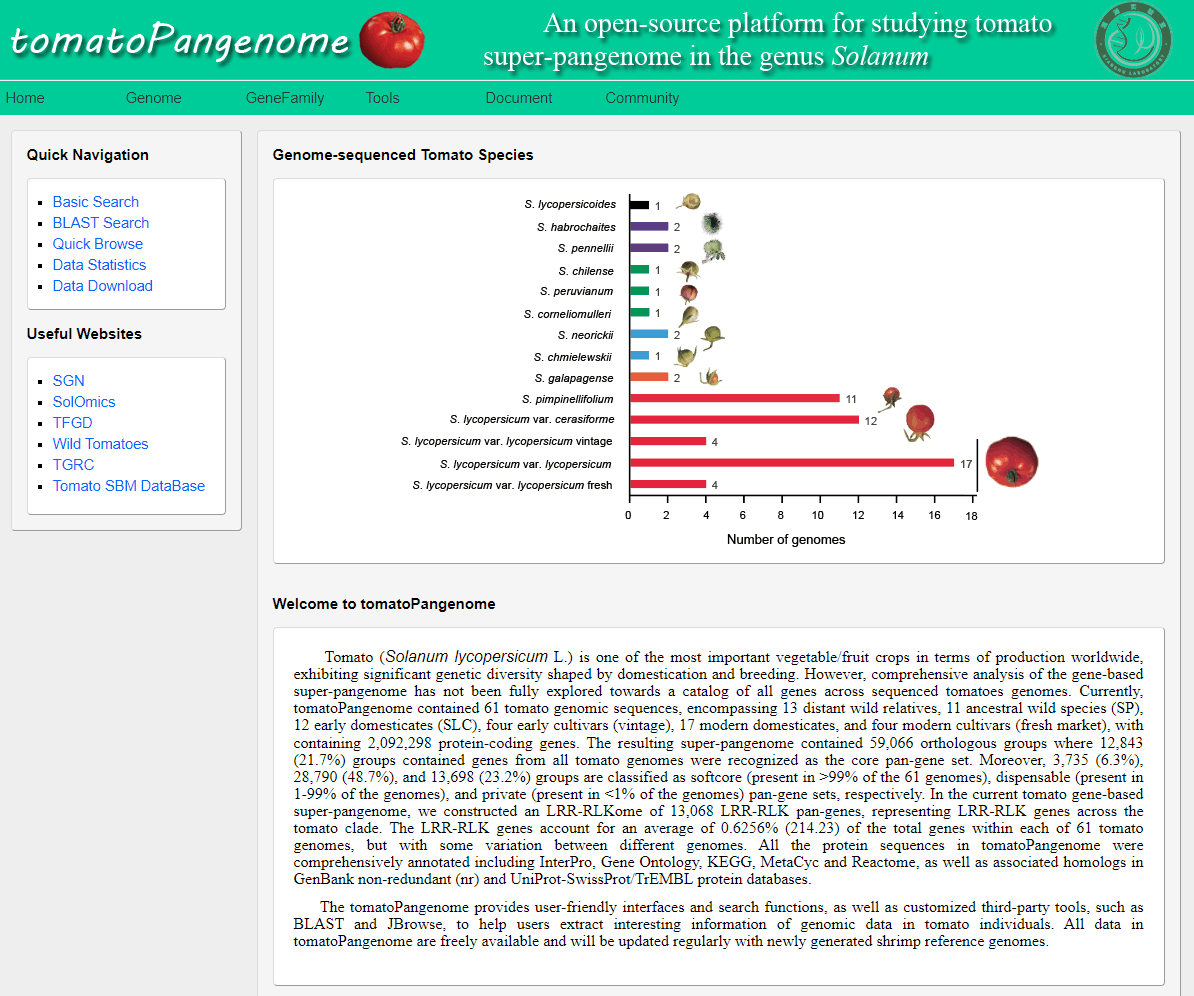


**Supplemental figure S6.** Homepage of tomatoPangenome platform.
